# Severe pediatric asthma therapy: Dupilumab

**DOI:** 10.3389/fped.2022.963610

**Published:** 2022-11-22

**Authors:** Giuliana Ferrante, Laura Tenero, Michele Piazza, Giorgio Piacentini

**Affiliations:** ^1^ Department of Surgical Sciences, Dentistry, Gynecology and Pediatrics, Pediatric Division, University of Verona, Verona, Italy; ^2^ Pediatric Division, University Hospital of Verona, Verona, Italy

**Keywords:** asthma, children, monoclonal antibody, IL-4, IL-13, asthma therapy, dupilumab

## Abstract

Severe asthma is a rare disease affecting <5% of children with asthma. This group of patients account for about 50% of the costs of healthcare for children with asthma. Nowadays, several biological agents are available for pediatric severe asthma. One of these is dupilumab, a monoclonal antibody against the Interleukin (IL)-4 receptor α-subunit that acts as an antagonist against both IL-4 and IL-13. Dupilumab binds the subunit of the IL-4 receptor, at the level of the subunit shared by the IL-13 receptor, blocking the inflammatory cascade of these two cytokines and the progression of the Th2-inflammatory pathway. The efficacy and safety of dupilumab have been investigated in recently published randomized controlled trials including pediatric patients with asthma. Currently, its use in asthma is approved in adults, adolescents, and children with severe asthma with type 2 inflammation, that are not controlled in spite of high-dose inhaled corticosteroids plus another maintenance drug. Studies are warranted for the evaluation of long-term treatment with dupilumab, including steroid sparing effect and discontinuation of treatment. Further research should also be planned in order to investigate dupilumab potential ability to interfere with the natural history of atopy since early childhood.

## Introduction

Asthma is a chronic disease characterized by reversible airflow obstruction, hyper-responsiveness, remodeling and progressive deterioration of lung function.

Severe asthma is defined as deficient control of asthma symptoms despite a therapy with high doses of inhaled corticosteroids (ICS) and long-acting β2-agonists (LABA) or need of recurrent oral corticosteroids (OCS) (level 4–5 GINA guidelines) (1) or by loss of asthma control when reducing the high-intensity treatment (2).

In the pediatric field, severe asthma is a rare pathology affecting <5% of children with asthma (3). Despite the low number of children with severe asthma, this group of patients account for about 50% of the costs of healthcare for children with asthma. Therefore, these patients are a challenge related to ample diagnostic evaluation and high consumption of healthcare sources.

It is firstly mandatory to confirm the diagnosis and evaluate the patient for at least three months to define severe asthma and the specific endotype of the patient.

According to the international guidelines (4), the evaluation of pediatric patient with severe asthma is based on the optimization of the standard therapy. Furthermore, the use of biological drugs must be considered when symptoms are not controlled, despite all the measures suggested (control of the environment factors and rigorous adhesion to drug therapy). Then, it is important to correctly select suitable patients for a specific biologic therapy, both for medical reasons and the high cost of these drugs.

Pediatric asthma is mainly characterized by a T helper type (Th)-2-inflammation in which the release of interleukin (IL)-4, IL-13, IL-5, and the immunoglobulin E (IgE) production increase eosinophilic survival.

In the last decades, many advances have been made in knowledge of pediatric asthma diseases and the role of the Th2-mediated inflammatory response.

Nowadays, several biological agents are now available for pediatric severe asthma. The approved biological drugs for the treatment of uncontrolled severe asthma target specific points of the Th2-inflammatory cascade and different agents target different endotypes of disease.

Their mechanism of action acts on peculiar cytokines of the Th2-inflammation cascade, such as IL-4, IL-5, and IL-13, and IgE, inhibiting definite targets in patients that do not respond to traditional therapy modifying the natural course of allergic inflammatory response (5).

One of these biological agents is dupilumab, a monoclonal antibody directed against the IL-4 receptor α-subunit (IL-4Ra) that acts as an antagonist against both IL-4 and IL-13 and is approved for pediatric severe type 2 asthma.

## Mechanisms of dupilumab

“Th2-mediated diseases” such as allergic asthma, atopic dermatitis (AD), allergic rhinitis (AR), chronic rhinosinusitis with nasal polyps (CRSwNP), and eosinophilic esophagitis (EoE) are characterized by type 2 inflammation associated to different pro-inflammatory cytokines released by epithelial cells (6–8).

These groups of inflammatory disorders that involved different tissues share the same mechanisms of action driven by CD4+ Th2 lymphocytes and type 2 innate lymphoid cells (ILC2). Inside that inflammatory pathway, IL-4 and IL-13 are produced by eosinophils, basophils, mast cells, CD8+ cells, and natural killer (NK) cells and have a key role in the allergic inflammatory response. Airways inflammation and remodeling are the typical asthma features related to these mediators.

IL-4 and IL-13 are involved in the pathophysiology as evidenced by the high level of these cytokines in peripheral blood, bronchoalveolar lavage (BAL), induced sputum, and bronchial mucosa of asthmatic subjects.

The polymorphisms found in the RAD50-IL-13 region of chromosome 5q31.1 of IL-13 is involved in determining the individual predisposition to asthma (9).

IL-4 and IL-13 play separate pathophysiologic functions in asthma. IL-4 acts in the initial polarization of naïve CD4+ Th cells to a Th2 phenotype, while IL-13 is essential in the bronchial hyperresponsiveness and in promoting airway inflammation and remodeling.

The heterodimeric IL-4 complex is composed by a common subunit called IL-4Rα, which pairs with subunits that mediate the action of IL-4 and IL-13 in different tissues (10).

The “subtype I” is expressed in hematopoietic cells and binds only—IL-4 to form IL-4R type 1. The first step starts when IL-4 binds the subunit IL-4Rα with high affinity. The complex IL-4/IL-4Rα is identified by the γ-chain and the IL-4 signaling is activated 10.

The “subtype II” is expressed in hematopoietic cells and non-hematopoietic cells and can be derived from the union of IL-4Rα with the IL-13 (IL-13Rα1) receptor to form a heterodimer between IL-13 and IL-4.

The connection between IL-4 and IL-13 to their receptor triggers the transduction of the signal by transphosphorylation and activation of the receptor subunit associated with Janus family protein kinase (JAK). In particular, receptor type I interacts with Janus kinases 1 (JAK1), 2 (JAK2), and 3 (JAK3), which are combined to the IL-4Rα, IL-13Rα1, and γc chains, respectively. JAK cascade induces the release of transcription factors and specific tyrosine residues located in the cytoplasmatic domain of the IL-4Rα (11, 12).

JAK1 and JAK3 phosphorylate specific tyrosine, which can then act as reduction sites for signal transducer for both activation of transcription (STAT)-6 and for insulin receptor substrate-2 (IRS-2) proteins.

After JAK1/JAK3-dependent tyrosine phosphorylation, STAT-6 dimerizes and move to the nucleus, where it upregulates the transcription factor GATA3. Then the binding to the promoter regions of the IL-5 and IL-13 genes improves their expression. IRS-2 proteins cooperate with the p85 subunit of phosphoinositide-3 kinase (PI3K) and with the adaptor protein growth factor receptor-bound protein 2 (Grb2), which are related to the PI3K/AKT pathway characterized by the proliferation of Th2 cells and differentiation of M2 macrophages. The heterodimeric IL-4Rα/IL-13Rα1 type II receptor complex is functionally correlated to JAK1/2, tyrosine kinase 2 (Tyk2), and STAT-6, but not to JAK3 and IRS-2 (13, 14).

IL-13 also binds with high affinity to the α2 chain of the IL-13 receptor (IL-13Rα2) implementing an endogenous self-regulating negative circuit that limits IL-13 activities (15, 16).

Furthermore, other pathways involved in the regulation of allergic responses (insulin receptor substrate IRS1/2)/phosphoinositide 3-kinases (PI3K)/mTOR Complex 2 (mTORC2)/AKR mouse thymoma kinase (AKT), SHC/MAPK, and Src homology 2 domain-containing protein tyrosine phosphatase-1 (Shp-1)) are activated by the IL-4 receptor.

Receptor type II interacts with JAK1, JAK2, and the tyrosine kinase 2 (TYK2), which can turn on STAT6 but not IRS2.

IL-4Rs play a key role in the differentiation of Th2 cells and IgE switch in B cells through this complicated mechanism causing specific disease phenotypes and endotypes.

This pathway of action of the IL-4R axis makes it a crucial target for precision medicine therapies with the aim to limit allergic inflammation and intervene in disease chronicity. In children, this aspect is particularly important, in consideration that an early intervention could limit the evolution of the atopy march and the onset of chronic inflammation and tissue remodeling (17).

**Figure 1:**
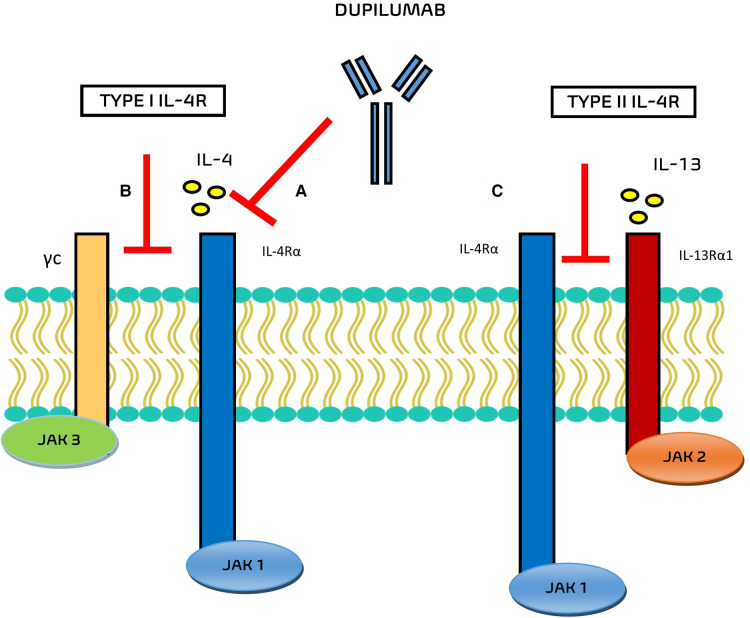
Mechanisms of action of Dupilumab. (**A**) Dupilumab inhibits IL-4 binding to IL-4Rα and/or (**B**) inhibits the recruitment of γc to IL-4Rα chain. (**C**) Dupilumab inhibits the recruitment of the IL-4Rα to IL-143Rα1.

In the field of monoclonal antibodies, dupilumab is an IgG monoclonal antibody that acts in this inflammatory pathway against the alpha subunit of the IL-4 receptor IL-4Rα (Figure 1), blocking the signal transduction pathways activated by both IL-4 and IL-13 in the IgE-mediated allergic inflammatory asthma.

The main ability of this monoclonal antibody is to bind the subunit of the IL-4 receptor, at the level of the subunit shared by the IL-13 receptor, blocking the inflammatory cascade of these two cytokines and the progression of the Th2-inflammatory pathway.

The interaction of dupilumab with the respective receptor complexes are still evaluated. Potential mechanisms of action of this drug include the inhibition of IL-4 binding to IL-4Rα, the inhibition of the recruitment of γc to IL-4Rα chain (for type I IL-4R), and the inhibition of the recruitment of the IL-4Rα to IL-13Rα1 (for type II IL-4R) (6).

At the present dupilumab is approved for treating adults, adolescents and children from the age of 6 years. In EU, EMA has also recently extended the use of dupilumab in TH2 severe asthma for treating adults, adolescents and children (age 6 years) characterized by high blood eosinophils and/or elevated fractional exhaled nitric oxide (FeNO) and symptoms not controlled by high-dose ICS plus another drug for maintenance treatment. GINA recommends dupilumab as an add-on treatment for patients (>12 years of age) with severe eosinophilic or type 2 asthma with blood eosinophilia (>300 cells/µl), high FeNO values (>20 ppb), uncontrolled respiratory symptoms, despite high-dose ICS plus long-acting ß2-agonist (LABA) or OCS.

The drug is available as a subcutaneous injection in prefilled syringes and is administered 400 mg once, then 200 mg every 2 weeks.

In patients with OCS dependent-asthma or for patients with severe asthma and comorbidity (moderate to severe atopic dermatitis or severe chronic rhinosinusitis with nasal polyposis) a subcutaneous starting dose of 600 mg (two 300 mg for injections), followed by 300 mg given every other week are recommended.

## Efficacy and safety of dupilumab in pediatric severe asthma

The efficacy and safety of dupilumab have been investigated in recently published randomized controlled trials (RCTs) including pediatric patients with asthma (Table 1).

**Table 1:** Summary of Dupilumab RCTs including pediatric patients with asthma.

Author	Study population	Intervention	Summary of main results
Rabe et al. (17) (LIBERTY ASTHMA VENTURE)	210 patients older than 12 years with severe OCS-dependent asthma	Add-on dupilumab (at a dose of 300 mg) every 2 weeks for 24 weeks or placebo for 24 weeks	↓ OCS use ↓ annualized severe exacerbation rate ↑ FEV_1_
Castro et al. (18) (LIBERTY ASTHMA QUEST)	1,902 patients aged 12 years or older with uncontrolled asthma	Add-on dupilumab (at a dose of 200 or 300 mg every 2 weeks) or placebo for 52 weeks	↓ annualized severe exacerbation rate (greater efficacy in participants with blood eosinophil concentrations >300 cells/µl) ↑ FEV_1_
Corren et al. (19) (LIBERTY ASTHMA QUEST)	1,083 patients aged 12 years or older with and 819 without uncontrolled allergic asthma	Add-on dupilumab (at a dose of 200 or 300 mg every 2 weeks) or placebo for 52 weeks	↓ annualized severe exacerbation rate ↑ FEV_1_ ↑ asthma control ↓ type 2 inflammatory biomarkers
Busse et al. (20) (LIBERTY ASTHMA QUEST)	814 patients aged 12 years or older with uncontrolled, moderate-to-severe asthma and comorbid perennial allergic rhinitis	Add-on dupilumab (at a dose of 200 or 300 mg every 2 weeks) or placebo for 52 weeks	↓ annualized severe exacerbation rate ↑ FEV_1_ (greater efficacy in patients with baseline blood eosinophil counts ≥300 cells/µl and FeNO >25 ppb) ↑ asthma control ↑ rhinoconjunctivitis health-related quality of life
Maspero et al. (21) (LIBERTY ASTHMA QUEST)	107 patients aged 12–17 years treated with medium-to-high-dose ICS plus one or two controllers	Add-on dupilumab (at a dose of 200 or 300 mg every 2 weeks) or placebo for 52 weeks	↑ FEV_1_ (greater efficacy in patients with baseline elevated baseline type 2 biomarker levels treated with dupilumab 200 mg) ↓ annualized severe exacerbation rate in patients treated with dupilumab 200 mg ↓ type 2 inflammatory biomarkers
Bacharier et al. (22) (LIBERTY ASTHMA VOYAGE)	408 children aged 6-11 years with uncontrolled moderate-to-severe asthma (two primary efficacy populations: patients with baseline blood eosinophil counts ≥150 cells/µl and FeNO ≥20 ppb and patients with baseline blood eosinophil counts ≥300 cells/µl)	Add-on dupilumab (at a dose of 100 or 200 mg every 2 weeks) or placebo every 2 weeks for 52 weeks	↓ annualized severe exacerbation rate ↑ FEV_1_ ↑ asthma control

FeNO, fractional exhaled nitric oxide; FEV_1_, forced expiratory volume in 1 s; ICS, inhaled corticosteroid; OCS, oral corticosteroid.

In the phase 3 trial LIBERTY ASTHMA VENTURE, 210 patients aged >12 years with severe OCS-dependent asthma were randomized to placebo or to add-on dupilumab 300 mg every 2 weeks for 24 weeks. The dupilumab group showed a percentage change in the OCS dose of −70.1%, in comparison with −41.9% in the group assigned to placebo (*p* < 0.001). Moreover, patients treated with dupilumab showed a severe exacerbation rate 59% [95% confidence interval (CI), 37–74] lower and a forced expiratory volume in 1 s (FEV_1_) 0.22 L (95% CI, 0.09–0.34) higher than that in those assigned to placebo. Reactions at the injection site were more commonly observed in patients assigned to dupilumab than placebo (9% vs. 4%), as well as transitory blood eosinophilia (14% vs. 1%). However, it should be pointed out that adolescent data were not extrapolated from the overall results (18). In the phase 3 trial LIBERTY ASTHMA QUEST, 1,902 patients aged >12 years with not controlled asthma were randomly assigned (2:2:1:1) to receive add-on dupilumab (200 or 300 mg every 2 weeks) or placebo for 52 weeks. The rate of severe asthma exacerbations was 0.46 (95% CI, 0.39–0.53) in patients assigned to dupilumab 200 mg and 0.87 (95% CI, 0.72–1.05) in those assigned to placebo, therefore 47.7% lower (*p* < 0.001). Notably, in participants with blood eosinophil concentrations >300 cells/µl, the rate of exacerbation was 65.8% lower than in the group assigned to placebo. With regard to pulmonary function, FEV_1_ increased by 0.32 L in patients receiving dupilumab 200 mg and by 0.14 L in those receiving placebo (*p* < 0.001), at week 12. Overall, similar findings were observed in patients assigned to a dupilumab dose of 300 mg every 2 weeks. Hypereosinophilia was more commonly observed in the dupilumab study groups (4.1%) than in patients receiving placebo (0.6%), whereas conjunctivitis was observed in 2.3% of the patients receiving dupilumab and 3.3% of those receiving placebo (19). Of interest, Corren et al. assessed dupilumab's effect in QUEST patients with (*n* = 1,083) and without (*n* = 819) atopic asthma (total serum IgE ≥30 IU/ml and ≥1 perennial aeroallergen-specific IgE ≥0.35 kU/L at baseline), demonstrating its ability to reduce severe exacerbation rates, improve FEV_1_ and asthma control, and suppress type 2 biomarkers of inflammation in both the subgroups. Therefore, these results highlight the beneficial role of dupilumab in treating both allergic and nonallergic asthma patients (20). Furthermore, Busse et al. published a *post hoc* analysis of the phase 3 LIBERTY ASTHMA QUEST study evaluating the efficacy of dupilumab in patients with not controlled, moderate-to-severe asthma and perennial allergic rhinitis (PAR). Out of the 1,902 patients, 814 (42.8%) had PAR, i.e., an allergic rhinitis history and ≥1 perennial aeroallergen specific IgE level ≥0.35 kU/L at baseline. Dupilumab, 200 and 300 mg every 2 weeks, vs. placebo significantly decreased severe exacerbations rates by 32.2% and 34.6% (*p* < 0.05 for both) and increased FEV_1_ at week 12 by 0.14 L and 0.18 L (*p* < 0.01 for both). Even higher efficacy was reported in patients with high blood eosinophil counts (≥300 cells/µl) and FeNO (25 ppb) levels. Patients in the dupilumab group also showed improvement in asthma control and rhinoconjunctivitis-specific health-related quality of life. Finally, dupilumab suppressed type 2 biomarkers of inflammation during the 52-week study period. Therefore, the study demonstrated that dupilumab, acting on type 2 inflammation occurring in both conditions, may contribute to increase the control of asthma and comorbid PAR (21). Again, adolescent data were not reported separately in these studies. However, in a subgroup analysis on 107 patients aged 12–17 years treated with medium-to-high-dose ICS plus one or two controllers, a change from baseline in FEV_1_ at week 12 vs. placebo (*p* < 0.05) was reported for both 200 mg (0.37 L; 95% CI, 0.13–0.61; *p* = 0.003) and 300 mg (0.27 L; 95% CI, 0.02–0.52; *p* = 0.037). Interestingly, in the majority of adolescents with high levels of type 2 inflammatory biomarkers assigned to dupilumab 200 mg, such improvement was even greater (0.43 L; 95% CI, 0.17–0.69; *p* = 0.002) than in the matched intention-to-treat adolescent subgroup. Furthermore, a 46% reduction in adjusted severe exacerbation rate (95% CI, 0.24–1.21) was reported in the dupilumab 200 mg subgroup vs. placebo. Nonetheless, the adjusted severe exacerbation rate in the dupilumab 300 mg subgroup was 13% higher than in the placebo subgroup. This may be ascribed to the imbalanced number of severe exacerbations reported in the past 12 months between the dupilumab 300 mg and the placebo subgroups (mean 1.53 and 2.22, respectively) that likely affected the adjusted rate of exacerbations. Indeed, the unadjusted severe exacerbation rate was numerically lower in both dupilumab subgroups vs. placebo in the overall population, as well as in adolescents with high levels of type 2 biomarkers. Finally, dupilumab reduced levels of type 2 biomarkers such as FeNO and serum total IgE and was overall well tolerated, supporting its use in the adolescent population (22).

More recently, in the LIBERTY ASTHMA VOYAGE, a 52-week phase 3 study, 408 children aged 6–11 years with not controlled moderate-to-severe asthma were randomized to receive add-on dupilumab (100 mg if weight ≤30 kg and 200 mg if weight >30 kg) or placebo every 2 weeks. In patients with the type 2 inflammatory phenotype (≥150 blood eosinophils/µl or FeNO levels ≥20 ppb at baseline), the annualized severe asthma exacerbations rate was 0.31 (95% CI, 0.22–0.42) in the dupilumab group and 0.75 (95% CI, 0.54–1.03) in the placebo group (relative risk reduction in the dupilumab group, 59.3%; 95% CI, 39.5–72.6; *p* < 0.001). The mean change from baseline in the predicted prebronchodilator FEV_1_ was 10.5 ± 1.0 percentage points with dupilumab and 5.3 ± 1.4 percentage points with placebo (mean difference, 5.2 percentage points; 95% CI, 2.1–8.3; *p* < 0.001). Additionally, dupilumab significantly improved asthma control with respect to placebo (*p* < 0.001). Similar findings were reported in the subgroup of patients with an eosinophil count ≥300 cells/µl at baseline. In both the two subgroups dupilumab generally showed an adequate safety profile in children, similarly to those reported in adults and adolescents. In particular, the most common adverse event in the dupilumab group was viral upper respiratory tract infection (12.2% vs. 9.7% in the placebo group). Eosinophilia was observed in 5.9% and 0.7% of the patients assigned to dupilumab and to placebo, respectively. However, most episodes were transitory laboratory findings with no associated symptoms. Mild parasitic infections were reported in 2.6% of the patients in the dupilumab group. Hospitalization due to asthma exacerbations were reported only in the dupilumab group (1.5%). The incidence of conjunctivitis was low in both groups and one case of keratitis was observed in each group (23). Additionally, within the LIBERTY ASTHMA VOYAGE study dupilumab was found to quality of life in children with type 2 asthma (24) and their caregivers (25); allergic rhinitis (AR)- health-related quality of life also improved in patients with comorbid AR (26).

With regard to the efficacy of biological treatment options in uncontrolled persistent asthma, very recently an indirect treatment comparison of dupilumab vs. each of the anti-IL-5 (benralizumab, mepolizumab, and reslizumab) and anti-IgE (omalizumab) therapies was conducted. The analysis included fourteen RCTs in patients aged 12 years and older. In the matched dupilumab subgroups annualized severe exacerbation rates were significantly reduced in comparison with benralizumab, mepolizumab, and reslizumab (54%, 28%, and 38%, respectively). Moreover, dupilumab was associated with significantly greater increase in FEV_1_ in comparison with benralizumab and reslizumab (at week 24) and omalizumab (at week 52). Hence, in this study dupilumab significantly reduced asthma exacerbation rates and was associated with greater improvements in pulmonary function than anti-IL-5s and omalizumab (27). These findings are in agreement with those of a previously published Cochrane intervention review assessing the efficacy and safety of anti-IL-13 or anti-IL-4 agents, in comparison with placebo, anti-IgE or anti-IL-5 agents, for the treatment of patients with asthma. Four studies evaluating dupilumab were included. In comparison with placebo, anti-IL-13/-4 agents were associated with a decrease in exacerbations that needed hospitalization or emergency department visit, in spite of increased adverse events, whereas no significant improvements in health-related quality of life and asthma control were observed. However, only four studies recruited children and adolescents, so participants in this age group accounted for less than 5% and therefore this review's results should be interpreted with caution for the pediatric population (28).

Safety of biological therapies is a major concern for clinicians dealing with the pediatric population. Due to the short-to-medium-term duration of studies on children and adolescents, evidence on the safety of dupilumab in the treatment of pediatric asthma is still limited. In particular, data for long-term use are required to estimate the risk of long-term adverse events/side effects. At present, there is no information about clinical significance and consequences of increases in peripheral blood eosinophils. When IL-13 is blocked by dupilumab, eosinophils migration is blocked. Therefore, increased eosinophilia can be considered consequent to IL-4/IL-13R blockade. Wechsler et al. recently conducted extensive *post hoc* analyses of 6,642 adults and adolescents who participated in dupilumab randomized, double-blind placebo-controlled trials, reporting transient increases in mean eosinophil counts in dupilumab-treated patients with asthma that were rarely associated with clinical symptoms (29). Nonetheless, concern has arisen about this potential adverse event and eosinophil-associated inflammation in other organs. Besides eosinophilic asthma, eosinophils may drive the inflammatory cascade underlying immunological hypereosinophilic conditions characterized by multiple-organ involvement, e.g., eosinophilic granulomatosis polyarthritis and hypereosinophilic syndrome. Although rare in children, these diseases should be considered in managing patients eligible for dupilumab with baseline elevated eosinophils. For this reason, it is advisable that a basal complete blood count must be included in the initial evaluation of patients affected by asthma and eligible for dupilumab, as well as rule out common parasitosis in case of basal elevated eosinophil count. With regard to conjunctivitis, a recent study evaluated the incidence and severity of conjunctivitis in dupilumab clinical trials involving adolescents with moderate-to-severe atopic dermatitis or uncontrolled asthma reporting no significant differences between the dupilumab and placebo groups, in contrast to the findings in patients with atopic dermatitis (30).

## Conclusion

Dupilumab is a biological drug with proven efficacy and reassuring safety profile in patients with type 2 inflammatory diseases, such as asthma. Currently, its use in asthma is approved by FDA and EMA in adults, adolescents, and children affected by severe asthma with type 2 inflammation characterized by high levels of eosinophils and/or FeNO, which is ineffectively controlled with high-dose ICS plus another maintenance drug. Additional studies are warranted for the evaluation of long-term treatment with dupilumab, including steroid sparing effect and discontinuation of treatment. Further research should also be planned in order to investigate the effect of dupilumab in children with asthma and comorbid conditions, as well as its potential ability to interfere with the natural history of atopy since early childhood. Moreover, data for long-term use are required to estimate the risk of long-term adverse events/side effects.

## References

[1] 2022 GINA Report, Global Strategy for Asthma Management and Prevention. Available from: https://ginasthma.org/gina-reports/ (AccessedMay 31, 2022).

[2] Agache I Beltran J Akdis C Akdis M Canelo-Aybar C Canonica GW Efficacy and safety of treatment with biologicals (benralizumab, dupilumab, mepolizumab, omalizumab and reslizumab) for severe eosinophilic asthma. A systematic review for the EAACI guidelines—recommendations on the use of biologicals in severe asthma. Allergy. (2020) 75(5):1023–42. 10.1111/all.14221 32034960

[3] Santos-Valente E Buntrock-Döpke H Abou Taam R Arasi S Bakirtas A Lozano Blasco J Biologicals in childhood severe asthma: the European PERMEABLE survey on the status quo. ERJ Open Res. (2021) 7(3):00143–2021. 10.1183/23120541.00143-2021 34409097 PMC8365152

[4] Bateman ED Hurd SS Barnes PJ Bousquet J Drazen JM FitzGerald JM Global strategy for asthma management and prevention: GINA executive summary. Eur Respir J. (2008) 31:143–78, Eur Respir J. (2018) 51(2). 10.1183/09031936.00138707 18166595

[5] Tenero L Piacentini G. New opportunities with biologic treatments in pediatric allergic and respiratory diseases. Pediatr Allergy Immunol. (2022) 33(Suppl 27):8–10. 10.1111/pai.13617 35080293 PMC9305856

[6] Licari A Castagnoli R Marseglia A Olivero F Votto M Ciprandi G Dupilumab to treat type 2 inflammatory diseases in children and adolescents. Paediatr Drugs. (2020) 22(3):295–310. 10.1007/s40272-020-00387-2 32157553

[7] Wynn TA. Type 2 cytokines: mechanisms and therapeutic strategies. Nat Rev Immunol. (2015) 15(5):271–82. 10.1038/nri3831 25882242

[8] Gandhi NA Pirozzi G Graham NMH. Commonality of the IL-4/IL-13 pathway in atopic diseases. Expert Rev Clin Immunol. (2017) 13(5):425–37. 10.1080/1744666X.2017.1298443 28277826

[9] Li X Howard TD Zheng SL Haselkorn T Peters SP Meyers DA Genome-wide association study of asthma identifies RAD50-IL13 and HLA-DR/DQ regions. J Allergy Clin Immunol. (2010) 125(2):328–35.e11. 10.1016/j.jaci.2009.11.018 20159242 PMC2824608

[10] Nelms K Keegan AD Zamorano J Ryan JJ Paul WE. The IL-4 receptor: signaling mechanisms and biologic functions. Annu Rev Immunol. (1999) 17:701–38. 10.1146/annurev.immunol.17.1.701 10358772

[11] Wills-Karp M Finkelman FD. Untangling the complex web of IL-4- and IL-13-mediated signaling pathways. Sci Signal. (2008) 1(51):pe55. 10.1126/scisignal.1.51.pe55 19109238 PMC4446705

[12] Gour N Wills-Karp M. IL-4 and IL-13 signaling in allergic airway disease. Cytokine. (2015) 75(1):68–78. 10.1016/j.cyto.2015.05.014 26070934 PMC4532591

[13] Pelaia C Vatrella A Gallelli L Terracciano R Navalesi P Maselli R Dupilumab for the treatment of asthma. Expert Opin Biol Ther. (2017) 17(12):1565–72. 10.1080/14712598.2017.1387245 28990423

[14] Chiba Y Goto K Misawa M. Interleukin-13-induced activation of signal transducer and activator of transcription 6 is mediated by an activation of Janus kinase 1 in cultured human bronchial smooth muscle cells. Pharmacol Rep. (2012) 64(2):454–8. 10.1016/S1734-1140(12)70788-0 22661199

[15] Zheng T Liu W Oh SY Zhu Z Hu B Homer RJ IL-13 receptor alpha2 selectively inhibits IL-13-induced responses in the murine lung. J Immunol. (2008) 180(1):522–9. 10.4049/jimmunol.180.1.522 18097054

[16] Harb H Chatila TA. Mechanisms of dupilumab. Clin Exp Allergy. (2020) 50(1):5–14. 10.1111/cea.13491 31505066 PMC6930967

[17] Tenero L Rossignoli S Piacentini G. Severe asthma: when to resort to biological agents. Pediatr Allergy Immunol. (2020) 31(Suppl 24):37–9. 10.1111/pai.13162 32017206

[18] Rabe KF Nair P Brusselle G Maspero JF Castro M Sher L Efficacy and safety of dupilumab in glucocorticoid-dependent severe asthma. N Engl J Med. (2018) 378(26):2475–85. 10.1056/NEJMoa1804093 29782224

[19] Castro M Corren J Pavord ID Maspero J Wenzel S Rabe KF Dupilumab efficacy and safety in moderate-to-severe uncontrolled asthma. N Engl J Med. (2018) 378(26):2486–96. 10.1056/NEJMoa1804092 29782217

[20] Corren J Castro M O’Riordan T Hanania NA Pavord ID Quirce S Dupilumab efficacy in patients with uncontrolled, moderate-to-severe allergic asthma. J Allergy Clin Immunol Pract. (2020) 8(2):516–26. 10.1016/j.jaip.2019.08.050 31521831

[21] Busse WW Maspero JF Lu Y Corren J Hanania NA Chipps BE Efficacy of dupilumab on clinical outcomes in patients with asthma and perennial allergic rhinitis. Ann Allergy Asthma Immunol. (2020) 125(5):565–76.61. 10.1016/j.anai.2020.05.026 32474156

[22] Maspero JF FitzGerald JM Pavord ID Rice MS Maroni J Rowe PJ Dupilumab efficacy in adolescents with uncontrolled, moderate-to-severe asthma: LIBERTY ASTHMA QUEST. Allergy. (2021) 76(8):2621–4. 10.1111/all.14872 33905544 PMC8360078

[23] Bacharier LB Maspero JF Katelaris CH Fiocchi AG Gagnon R de Mir I Dupilumab in children with uncontrolled moderate-to-severe asthma. N Engl J Med. (2021) 385(24):2230–40. 10.1056/NEJMoa2106567 34879449

[24] Fiocchi A Phipatanakul W Durrani SR Cole J Mao X Msihid J Dupilumab improves asthma control and quality of life in children with uncontrolled persistent asthma. Eur Respir J. (2021) 58:PA3920. 10.1183/13993003.congress-2021.PA3920

[25] Fiocchi A Phipatanakul W Durrani SR Cole J Liu D Msihid J Dupilumab improves quality of life in caregivers of children with uncontrolled moderate-to-severe asthma: LIBERTY ASTHMA VOYAGE study. Pediatrics. (2022) 149:255.

[26] Fiocchi A Phipatanakul W Durrani SR Cole J Liu D Msihid J Dupilumab improves asthma control, and Health-Related Allergic Rhinitis-Related Quality of Life in children with uncontrolled persistent asthma with comorbid allergic rhinitis. J Allergy Clin Immunol Pract. (2022) 149(Issue 2, Supplement):AB135. 10.1016/j.jaci.2021.12.460

[27] Bateman ED Khan AH Xu Y Guyot P Chao J Kamat S Pairwise indirect treatment comparison of dupilumab versus other biologics in patients with uncontrolled persistent asthma. Respir Med. (2022) 191:105991. 10.1016/j.rmed.2020.105991 35090688

[28] Gallagher A Edwards M Nair P Drew S Vyas A Sharma R Anti-interleukin-13 and anti-interleukin-4 agents versus placebo, anti-interleukin-5 or anti-immunoglobulin-E agents, for people with asthma. Cochrane Database Syst Rev. (2021) 10(10):Cd012929. 10.1002/14651858.CD012929.pub2 34664263 PMC8524317

[29] Wechsler ME Klion AD Paggiaro P Nair P Staumont-Salle D Radwan A Effect of Dupilumab on blood eosinophil counts in patients with asthma, chronic rhinosinusitis with nasal polyps, atopic dermatitis, or eosinophilic esophagitis. J Allergy Clin Immunol Pract. (2022) 10(10):2695–709. 10.1016/j.jaip.2022.05.019 35636689

[30] Bansal A Simpson EL Paller AS Siegfried EC Blauvelt A de Bruin-Weller M Conjunctivitis in Dupilumab clinical trials for adolescents with atopic dermatitis or asthma. Am J Clin Dermatol. (2021) 22(1):101–15. 10.1007/s40257-020-00577-1 33481203 PMC7847457

